# Baseline levels of miR-223-3p correlate with the effectiveness of electroconvulsive therapy in patients with major depression

**DOI:** 10.1038/s41398-023-02582-4

**Published:** 2023-09-13

**Authors:** Lalit Kaurani, Matthias Besse, Isabel Methfessel, Aditi Methi, Jiayin Zhou, Ranjit Pradhan, Susanne Burkhardt, Laura Kranaster, Alexander Sartorius, Ute Habel, Michael Grözinger, Andre Fischer, Jens Wiltfang, David Zilles-Wegner

**Affiliations:** 1 Department for Epigenetics and Systems Medicine in Neurodegenerative Diseases, German Center for Neurodegenerative Diseases Goettingen, 37075 Goettingen, Germany; 2 Department of Psychiatry and Psychotherapy, University Medical Center Goettingen, 37075 Goettingen, Germany; 3 Department of Psychiatry, Vitos Klinikum Heppenheim, 64646 Heppenheim, Germany; 4 Department of Psychiatry and Psychotherapy, Central Institute of Mental Health, Medical Faculty Mannheim and University of Heidelberg, 68159 Mannheim, Germany; 5 Department of Psychiatry, Psychotherapy and Psychosomatics, RWTH Aachen University, 52074 Aachen, Germany; 6 Cluster of Excellence MBExC, University of Göttingen & University Medical Center Goettingen, 37075 Göttingen, Germany; 7 Clincal Science Group, German Center for Neurodegenerative Diseases (DZNE), 37075 Goettingen, Germany; 8 Neurosciences and Signaling Group, Institute of Biomedicine (iBiMED), Department of Medical Sciences, University of Aveiro, 3810-193 Aveiro, Portugal

**Keywords:** Epigenetics and behaviour, Predictive markers, Depression

## Abstract

There is a strong medical need to develop suitable biomarkers to improve the diagnosis and treatment of depression, particularly in predicting response to certain therapeutic approaches such as electroconvulsive therapy (ECT). MicroRNAs are small non-coding RNAs that have the ability to influence the transcriptome as well as proteostasis at the systems level. Here, we investigate the role of circulating microRNAs in depression and response prediction towards ECT. Of the 64 patients with treatment-resistant major depression (MDD) who received ECT treatment, 62.5% showed a response, defined as a reduction of ≥50% in the MADRS total score from baseline. We performed smallRNA sequencing in blood samples that were taken before the first ECT, after the first and the last ECT. The microRNAome was compared between responders and non-responders. Co-expression network analysis identified three significant microRNA modules with reverse correlation between ECT- responders and non-responders, that were amongst other biological processes linked to inflammation. A candidate microRNA, namely miR-223-3p was down-regulated in ECT responders when compared to non-responders at baseline. In line with data suggesting a role of miR-223-3p in inflammatory processes we observed higher expression levels of proinflammatory factors *Il-6*, *Il-1b*, *Nlrp3* and *Tnf-α* in ECT responders at baseline when compared to non-responders. ROC analysis of confirmed the diagnostic power of miR-223-3p demarcating ECT-responders from non-responder subjects (AUC = 0.76, *p* = 0.0031). Our data suggest that miR-223-3p expression and related cytokine levels could serve as predictors of response to ECT in individuals with treatment-resistant depressive disorders.

## Introduction

Major depressive disorder (MDD) is a frequent and recurring mental illness that is associated with morbidity and mortality [1–4] affecting over 264 million individuals worldwide [5].

The initial line of treatment consists of psychotherapy, antidepressants and other drugs that increase antidepressant effect. Antidepressants are believed to exert their effects through triggering transcriptional and translational processes that result in neuroplastic alterations. Neurotrophic substances such as brain-derived neurotrophic factor (BDNF) and vascular endothelial growth factor (VEGF) may be, at least in part, responsible for these effects [6–8]. Despite the fact that antidepressants are generally effective in the treatment of MDD, 37% of patients do not respond within 6–12 weeks of medication, and 53% do not reach remission [9].

ECT is an effective treatment for severe and pharmaco-resistant depression, which is typically performed as a series of 10-12 treatments within 4 weeks [10, 11]. ECT utilizes controlled and safe electric currents to induce a generalized seizure while the patient is under anesthesia and muscle relaxation [12]. The mechanism of action is thought to, among others, involve neurotrophic processes affecting and normalizing cerebral networks [13]. To further understand the mechanisms underlying ECT in more detail and to find markers that could help predicting the effect of ECT in the single patient is of high clinical importance.

MicroRNAs (miRs) are small non-coding RNA molecules with length of 21–23 nucleotides [14]. MiRs regulate gene expression and protein production by interacting with messenger RNA (mRNA) transcripts, leading to their degradation or inhibition of translation [15]. A single miR can influence hundreds of transcripts, making miRNAs appealing targets for various disorders including depression [16–18]. MiRs can also function in a paracrine manner and contribute to inter-organcommunication [19–22]. Therefore, miRs are discussed as promising biomarker for brain diseases, since alterations of miRs expression in liquid biopsies such as blood may reflect alterations in the brain. In line with this, there is increasing evidence that miRs are deregulated in the blood and in brain tissue of patients suffering from neuropsychiatric diseases [23–25].

The current study aimed to investigate the effects of ECT on miR expression in the peripheral blood of patients with severe and pharmaco-resistant MDD. Our data indicate that circulating levels of miR-223–3p in MDD patients at baseline can predict the effectiveness of ECT treatment. In agreement with this observation, we identified that the miR-223–3p targets NLRP3 and IL-6 which also show differential expression in ECT responders versus non-responders. In summary, our data further elucidate the mechanisms underlying ECT and suggest that the analysis of miR-223–3p and its targets in blood can help to predict treatment response.

## Materials and methods

### Human subjects

Written informed consent was obtained from all participants prior to study participation. The study was approved by the ethics committee of the University Medical Centre Göttingen (reference number 21/6/14). Patients were clinically diagnosed according to ICD-10 criteria. Depression was defined via the presence of all 3 main criteria (depressed mood, loss of interest/pleasure, loss of energy) and a minimum of 4 further criteria of depression. This clinical diagnosis is reflected by MADRS scores >34 in many patients from our sample. Pharmacological treatment resistance is defined as non-response to at least two different antidepressants or augmentation strategies. Concomitant medication was kept unchanged throughout the ECT treatment course. Blood was drawn at the baseline visit (visit 1) prior to the start of ECT as well as one day after the first and one day after the last ECT treatment. We performed analyses on 64 patients with MDD who participated in the study (40 ECT responders and 24 ECT non-responders). All individuals had their depressive symptoms confirmed using the Montgomery–Åsberg Depression Rating Scale (MADRS). The procedure for blood collection from patients was approved by Universitätsmedizin Göttingen (UMG).

Blood was taken and processed according to the manufacturer’s protocol before being stored at -80C in PAXgene Blood RNA Tubes (PreAnalytix/Qiagen). The PAXgene tubes were thawed and incubated overnight at room temperature. RNA was extracted using PAXgene Blood RNA Kits according to the manufacturer’s protocol (Qiagen). RNA concentrations were measured by UV measurement. RNA integrity for library preparation was determined by analyzing them on an RNA 6000 NanoChip using a 2100 Bioanalyzer (Agilent Technologies). Patient characteristics including age, gender, and depression severity score are listed in Table 1 & supplementary table 1.

**Table 1:** MDD cohort.

	Total group (*n* = 64)	Responders (*n* = 40)	Non-responders (*n* = 24)
Age (years)	55.9 ± 16	58.825 ± 14.75	51.04 ± 17.12
Sex (f/m)	38/26	22/18	16/8
MADRS visit 1	32.6 ± 7.4	32.1 ± 7.45	33.54 ± 6.73
MADRS visit 3	14.5 ± 10.7	7.55 ± 10.8	27 ± 6.8
PSI	72.17 ± 12.58	69.17 ± 12.9	76.87 ± 12.02
ASEI	8858.05 ± 6376.3	6901.84 ± 4360.92	13022.94 ± 9924.84

Values are given as means and standard deviations (age, MADRS, PSI and ASEI) and frequencies (sex).

### Electroconvulsive therapy

Age based or titration-threshold dosing was used in the initial session, dosage was then adjusted depending on clinical response as well as seizure quality. PSI and ASEI index were used to quantify the quality of the induced seizure. Specifically, PSI stands for Postictal Suppression Index and is given in %, while ASEI refers to the Average Seizure Energy Index and is given in µV^2/1000. PSI evaluates post-seizure brain suppression, while ASEI assesses the overall seizure energy, both contributing to optimizing ECT protocols and outcomes. Electrode placement with right unilateral bitemporal, and left anterior right temporal position was chosen (and adjusted if required) according to response and tolerability. For anesthesia, methohexital, etomidate, esketamine, propofol and thiopental were used, and succinylcholine was used as muscle relaxant.

### Clinical assessment of depressive symptom severity

The Montgomery–Åsberg Depression Rating Scale (MADRS) was used to determine depression severity at baseline and following completion of the ECT treatment series. Response was defined as ≥50% reduction in MADRS score from baseline to post-treatment. Percent change in MADRS score from baseline to end-of-treatment was used as the continuous outcome measure [26, 27].

### High throughput smallRNA sequencing

MiRs can be quantified via smallRNA sequencing. In total, we sequenced 191 samples comprising all three time points of 64 study subjects (Supplementary table 1). One sample was not sequenced due to poor RNA quality. Using the NEBNext® smallRNA library preparation kit, we prepared smallRNA libraries from 150 ng total RNA per manufacturer’s instructions. Briefly, cDNA synthesis and PCR amplification were performed on total RNA. Libraries were size-selected using PAGE, and a 150 bp small RNAome band was purified and quantified. Libraries were sequenced at 2 nM concentration using the Illumina HiSeq 2000 platform with 50-bp single reads. Demultiplexing was done with CASAVA 1.8, adapters removed using Cutadapt-1.8.1, and sequence data quality assessed via FastQC (http://www.bioinformatics.babraham.ac.uk/projects/fastqc/). Quality metrics included read count, GC content, N content, sequence length distribution, duplication levels, overrepresented sequences, and Kmer content.

### Data processing and QC

Reads were aligned to the Homo_sapiens.GRCh38.p10 genome assembly (hg38) using miRdeep2 package (https://www.mdc-berlin.de/content/mirdeep2-documentation). Genome sequence were available from UCSC Genome Browser (https://genome.ucsc.edu/). Reads mapping were performed using Bowtie-build tool (version 1.12) using default option [28]. Perl based scripts available in MirDeep2 package were used for mapping and generating raw counts for miRs. Reads were mapped to hg38 using mapper.pl script. We discarded reads that were less than 18 nucleotides. These reads were further used for quantification of known miRs. To quantify known miRs quantifier.pl from miRDeep2 package was used. This step provided us with raw miRs counts. We generated raw counts for 64 subjects, resulting in 191 smallRNAseq datasets from baseline, V2 and V3 (Supplementary table 1; for one individual RNA isolation failed for V2). Next, prior to DE analysis, the raw read counts were log2-transformed and normalized for library size prior to differential expression analysis. We identified outliers using the WGCNA R package [29]. Due to the sensitivity of the WGCNA to outliers, the adjacency function of the WGCNA was used to generate a standardized connectivity score (Z). Low- quality samples (Z > 2.5 or Z -2.5) were identified as outliers and excluded from further analysis. On this basis, we removed 25 samples and analyzed 166 smallRNAseq datasets from 60 subjects (40 responders and 20 non-responders) from V1 (baseline), V2 and V3 (Supplementary table 3, V1 responder *n* = 38; V2 responder *n* = 36; V3 responder *n* = 39; V1 non-responder *n* = 16; V2 non-responder *n* = 18; V3 non-responder *n* = 19).

### Differential expression (DE) analysis

We used raw counts for the DE analysis. After removing poor quality samples (see Data processing and QC**)** we furthermore filtered the data for miRs that have at least 5 read counts in at least 50% of the studied samples. The RUVSeq package [30] was used to account for hidden batch effects and eliminate unnecessary variance. The data were corrected for age and gender. DESeq2 [31] was used to perform differential expression analysis. The cut-off criteria for selecting differentially expressed miRs were defined as followed: miRNAs must have a basemean of at least 100 reads, a false discovery rate (FDR) of 10% or less (i.e., adjusted *p*-value < 0.1), and a log_2_-fold change cut-off of ± 0.35. The adjusted *p*-value in DESeq2 analysis, also known as the FDR, is a statistical measure that corrects for multiple testing to reduce the chance of false positives in gene expression studies.

### WGCNA analysis

The weighted gene co-expression network analysis (WGCNA) package (version 1.70.3) in R (version 4.1.0) was used to analyze the microRNAome co-expression network [29]. We started by regressing the sequencing data to remove age, gender, and other latent factors. Following that, normalized counts were converted using the variance stabilizing transformation (VST). The transformed data was then used to compute pair-wise Pearson’s correlations between miRs s and define a co-expression similarity matrix, which was further transformed into an adjacency matrix. Then, using approximate scale-free topology, a soft-thresholding power of 7 was determined and utilized to calculate the pair- wise topological overlap matrix (TOM) and the corresponding dissimilarity values (1-TOM) between miRs in order to build a signed miRs co-expression network [32]. miRs s co-expressed with a minimum module size of 10 were discovered using the cutreeDynamic function with the following parameters: method = “hybrid”, deepSplit = 4, pamStage = T, pamRespectsDendro = F. Modules that were closely related were combined using a 0.25 dissimilarity correlation threshold. We evaluated the association between module eigenvalues and treatment response across three visits (V1, V2, V3) using a binary trait matrix, where rows represent samples and columns represent visits. Responders were assigned a value of 1 and non-responders a value of 0. Pearson’s correlation coefficients were calculated between module eigenvalues and binary traits for each visit. Positive correlations indicate microRNAs in a module are upregulated in responders or linked to a positive response, while negative correlations suggest downregulation or association with a negative response.

### Enriched gene ontology and pathways analysis

We constructed a Gene Regulatory Network (GRN) for miRNA-target genes using validated miRs targets from miRTarBase (v7.0) (http://mirtarbase.mbc.nctu.edu.tw/). Further filtering of the miRs target genes was performed based on their expression in the brain. The ClueGO v2.2.5 plugin for Cytoscape 3.2.1 was used to identify biological processes and their pathways in miRNA-target genes [33]. In the ClueGo plugin, the relevance of each term was determined using a two-sided hypergeometric test, and the *P* value was corrected using Benjamini-Hochberg method. The pathway analysis was performed using the KEGG (https://www.genome.jp/kegg/) and Reactome (https://reactome.org/) databases. Biological processes and pathways with an adjusted *p*-value of less than 0.05 were chosen for further investigation. PsyGeNET [34] database was used to select MDD and treatment-resistant genes (TRD) associated genes.

### Quantitative PCR experiment

cDNA synthesis was performed using the miScript II RT Kit (Qiagen) according to manufacturer protocol. In brief, 200 ng total RNA was used for cDNA preparation. HiFlex Buffer was used so that cDNA can be used for both mRNA and miRs quantitative PCR (qPCR). miRs specific forward primer and a universal reverse primer were used to quantification. The U6 small nuclear RNA gene was used as an internal control. For mRNA quantification, gene specific forward and reverse primers were used. The relative amounts of mRNA were normalized against internal control GAPDH. The fold change for each mRNA was calculated using the 2^–ΔΔCt^ method. Light Cycler® 480 Real-Time PCR System (Roche) was used to perform qPCR. Primer sequences are provided in supplementary table 2.

### Statistics and reproducibility

Statistical analyses were conducted using GraphPad Prism v8.2 or RStudio (v1.4.1106). We employed two-tailed unpaired t-tests for comparing independent group means, Pearson’s correlation coefficients for assessing relationships between MADRS scores and microRNA expression, and the GeneOverlap R package with Fisher’s exact test to analyze microRNA target gene overlaps with depression-related genes. Weighted co-expression microRNA module relationships in the adjacency matrix were also evaluated using Pearson’s correlation. Results are presented as boxplots or mean ± s.e.m., with boxplots displaying interquartile range (IQR), median, data range within 1.5x IQR, and potential outliers. The sample size (n) represents biological samples. Linear regression models were used to control for age and gender effects. We performed differential expression analysis using DESeq2. When comparing two groups with DESeq2, the Wald test was used for hypothesis testing. Enriched gene ontology and pathway analysis was performed using Fisher’s exact test followed by a Benjamini-Hochberg correction. An examination of receiver-operating characteristics (ROC) was performed to examine the diagnostic power for differentially expressed miRs at baseline. To account for changes in absolute expression values between responder subjects, a ROC analysis was performed using the z-scored miRNA expression values for differentially expressed miRNAs [35]. The area under the ROC curve (AUC) and accompanying *p* values were used to assess the diagnostic power of ECT- responder and non-responder separation.

## Results

### Clinical outcome

Patient characteristics are presented in Table 1 & Supplementary table 1. Of the 64 subjects, 62.5% responded to treatment, with a mean decrease in MADRS score of ≥50% (Supplementary table 1, Supplementary fig. 1A, B). Individuals had a mean number of 12 treatments. Supplementary table 1 summarizes the number of participants who finished treatment and underwent blood collection.

### Time course of changes in peripheral blood microRNAome during ECT

**Fig. 1:**
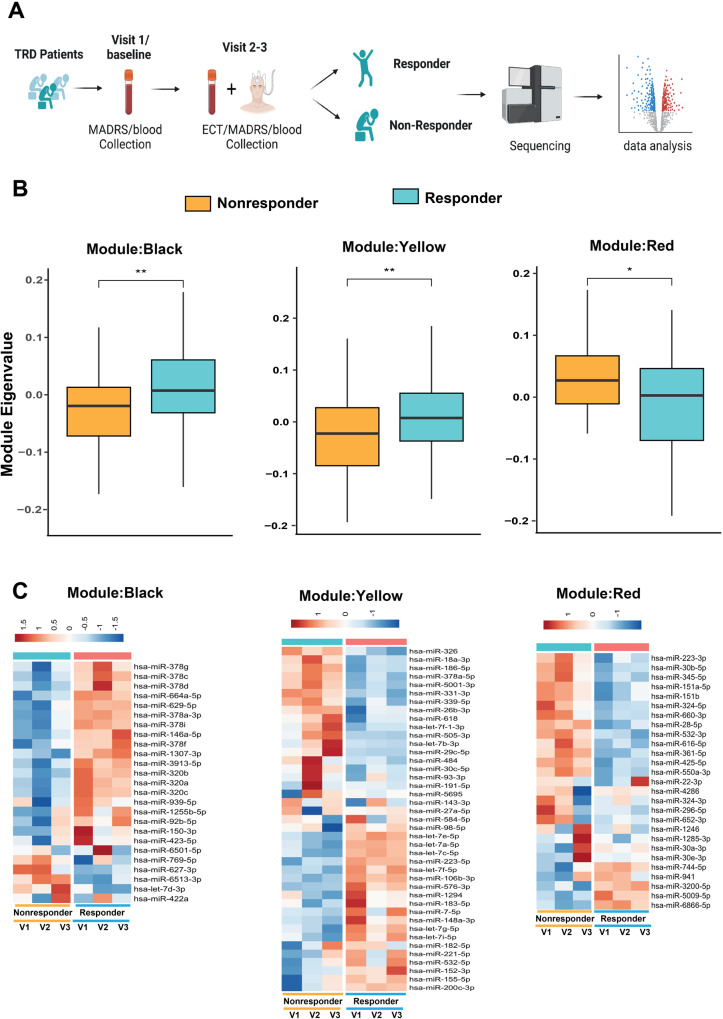
Co-expressing deregulated miR modules during the course of ECT. **A** This figure depicts study design. Blood was taken from each participant (64 subjects) at three different visits. Visit 1 represents baseline. Each participant was given electroconvulsive therapy (ECT) at visit two and three. After the treatment smallRNA sequencing was performed. After initial quality control of the sequencing dataset microRNAome was analyzed for 166 smallRNAseq dataset from 60 subjects. **B** Module eigengene values were plotted for black, yellow and red modules. The horizontal line in the box plot represents the median, the box spans 25 and 75% quantile, and the whiskers represent the smallest and largest values in the 1.5x interquartile range. **C** Heatmap shows the expression of co-expressed miRs in black, yellow and red modules among ECT-responder and non- responders for each visit. The intensity of the color represents the expression status. Red represents higher expression and blue represents reduced expression at each visit. (*P < 0.05; **P < 0.01, ***P < 0.001; ****P < 0.0001). Two-tailed unpaired *t*-test was performed to calculate significance.

We sequenced smallRNAs as we were interested in microRNAome changes during the time course of ECT (Fig. 1A). Sequencing was carried out in two distinct batches, and data were corrected for confounding factors including age and batch. Samples with poor sequencing quality were removed since not all samples passed quality control (QC) analysis. Thus, we were able to analyze 166 smallRNAseq datasets representing 35 ECT responders with a full dataset from baseline, V2 and V3. For 3 ECT-responders data was only available from 2 time points, while for 2 ECT-responders data was only available from one time point. Similarly, for 14 ECT non-responders smallRNAseq was available for all 3 time points, for 5 ECT non-responders data was available for only 2 time points, while for 1 ECT non-responders data was available for only one time point (Supplementary table 3). While all participants in our study received ECT, not all individuals responded to the treatment in the same way, which was measured by a reduction in the MADRS total score of ≥50% from baseline. To identify miRs associated with ECT treatment response, we compared their expression levels between ECT responders and non-responders at each time point. We employed WGCNA (weighted co-expression analysis) to identify co-expression modules associated with ECT treatment response, rather than comparing individuals longitudinally across different visits. Using WGCNA, we identified nine different co-expression modules (Fig. 1B, Supplementary fig. 2A, B). Pearson’s correlation coefficient was used to determine the relationship between module eigenvalues and ECT treatment at V1 (baseline) to V2 and V3 (Fig. 1A, Supplementary fig. 2A). The MEblack, MEyellow, and MEred modules correlated significantly with the course of ECT (adjusted *p*-value < 0.1; 10%FDR; Fig. 1B, Supplementary Fig. 2A; supplementary table 4). The MEblack module contained co-expressing miRs that were mainly upregulated in ECT responders, while the MEyellow module exhibited many miRs that were upregulated but also a substantial amount of miRs that were down-regulated. The MEred module contained co-expression miRs that were down- regulated in ECT responders (Fig. 1B, C).

**Fig. 2:**
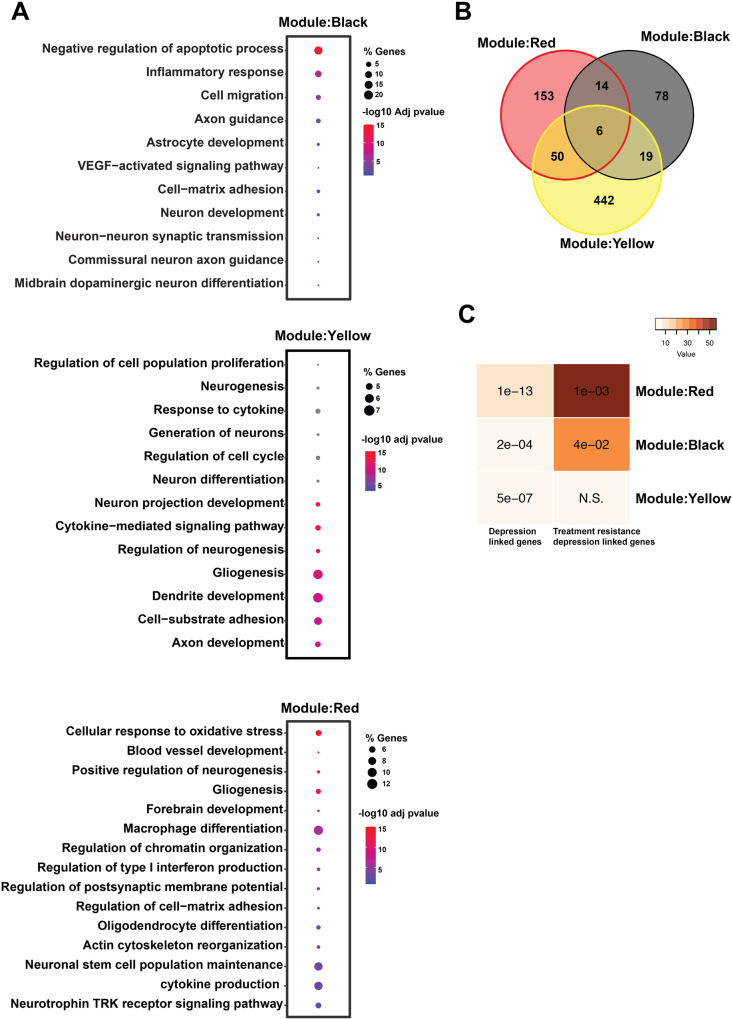
Changes in inflammatory response and synaptic plasticity are linked with ECT. **A** This figure depicts the selected deregulated biological processes targeted by co-expressed miRs of black, yellow and red modules. Selected deregulated biological processes are enriched for inflammation, VEGF signaling, synaptic assembly and axon guidance. **B** The overlap among target genes of co-expressed miR modules. **C** Hypergeometric overlap of the co-expressed miR module target genes with genes related with depression based on human genome-wide association studies and transcriptional-based study. Fisher’s exact test was performed, and the data are presented with a Benjamini Hochberg (BH) corrected *p*-value. A color map illustrates the significance and overlap strength. The PsyGeNEt database was utilized to identify genes associated with depression and TRD.

To gain insight into the biological processes related to the observed miRNA changes, we conducted Gene Ontology (GO) analysis on each significant module. The black module’s miRNAs were associated with inflammatory response, VEGF signaling, neuron-astrocyte development, and synaptic transmission. The yellow module’s miRNAs were enriched for processes involving oxidative stress, cytokine and interferon production, gliogenesis, and dendrite development. The red module’s miRNAs were involved in oxidative stress, neurogenesis, chromatin organization, and pathways linked to inflammatory processes such as type I interferon production (Fig. 2A). We identified 56 genes that were common between the red and yellow module, 25 genes between yellow and black module and 20 between red and black module. All three significantly co-expressed modules shared 6 common genes (*VEGFA, CDKN1A, IGF1R, PTEN, GSK3B, MALAT1*) (Fig. 2B). Subsequently, we compared each module’s miR-targeted genes to genes previously linked to MDD and treatment-resistant depression (TRD). Interestingly, the genes deregulated in all these miR modules significantly overlapped with depression disease associated genes (Fig. 2C). We identified that red and black module miR-target genes significantly overlapped with genes linked with TRD, while no significant overlap of the yellow module miRs target genes was found (Fig. 2C). In conclusion, these data further support the effect of ECT on genes and miRs important for synaptic plasticity, neuronal integrity and gliogenesis that are known to be deregulated in MDD and TRD.

### Effect of first treatment of ECT on microRNAome

Next, we performed differential expression analysis to investigate the impact on the microRNAome between ECT responders and non-responders after the first ECT treatment, hence at visit 2. Four miRNAs were significantly different between ECT responders and non-responders (basemean ≥ 100, log_2_ fold change ± 0.35, adjusted *p*-value < 0.1). It is important to note that the classification of responder and non-responder was based on the phenotyping data obtained after the last ECT treatment. MiR-144-5p, miR-320b and miR-320c had higher expression levels, while miR-142-3p showed lower expression in responders (Supplementary fig. 3A; Supplementary Table 5). We did not observe significant differences in the expression of these miRNAs at baseline. (Comparison at baseline, V1 responder vs. non-responder, DEseq2 Wald test: miR-144-5p, *P* = 0,95; miR-320b, *P* = 0,87; miR-320c *P* = 0,87; miR-142-3p, *P* = 0,55).

Target mining discovered 128 target transcripts for the 3 upregulated miRNAs. Enrichment analysis of these targets revealed GO biological process including those involved in neuroinflammation (TGF-beta signaling), neurodegeneration (oxidative phosphorylation), and brain development (neurotrophin signaling) (Supplementary fig. 3B). Pathways associated with neurodegeneration were significantly overrepresented. These findings suggest that after a single ECT treatment, neuroinflammation and oxidative stress-related biological processes were regulated in ECT responders.

We identified 286 potential target transcripts for the down-regulated miRNA miR-142-3p. The targets were enriched for GO biological processes involving neurotransmission (dopaminergic signaling), synaptic plasticity (long-term potentiation), and brain development (axon guidance, oxytocin signaling) (Supplementary fig. 3B). As miR down-regulation typically leads to increased target gene expression, we hypothesized that responder subjects may see improvement in neurotransmission and synaptic plasticity related processes after the first ECT treatment. Supplementary tables 6 and 7 provide a list of the enriched pathways and corresponding genes associated with deregulated miRs.

We examined the co-expression pattern of the differentially expressed miRs and found that they form clusters in miR modules. The upregulated miRs (miR-320b, miR-320c) were part of the MEblack module (Fig. 1C) which was enriched in pathways related to inflammation, VEGF signaling, neuron- astrocyte formation, and synaptic transmission. The MEblue module was associated with miR-142-3p and miR-144-5p (Supplementary table 4).

Our findings suggest that the first ECT treatment leads to significant changes in the microRNAome of whole blood in MDD patients. These changes may indicate similar improvements in neurotransmission and synaptic plasticity related processes in the CNS of responder subjects after ECT [36, 37].

### Changes in microRNAome expression following completion of ECT treatment

**Fig. 3:**
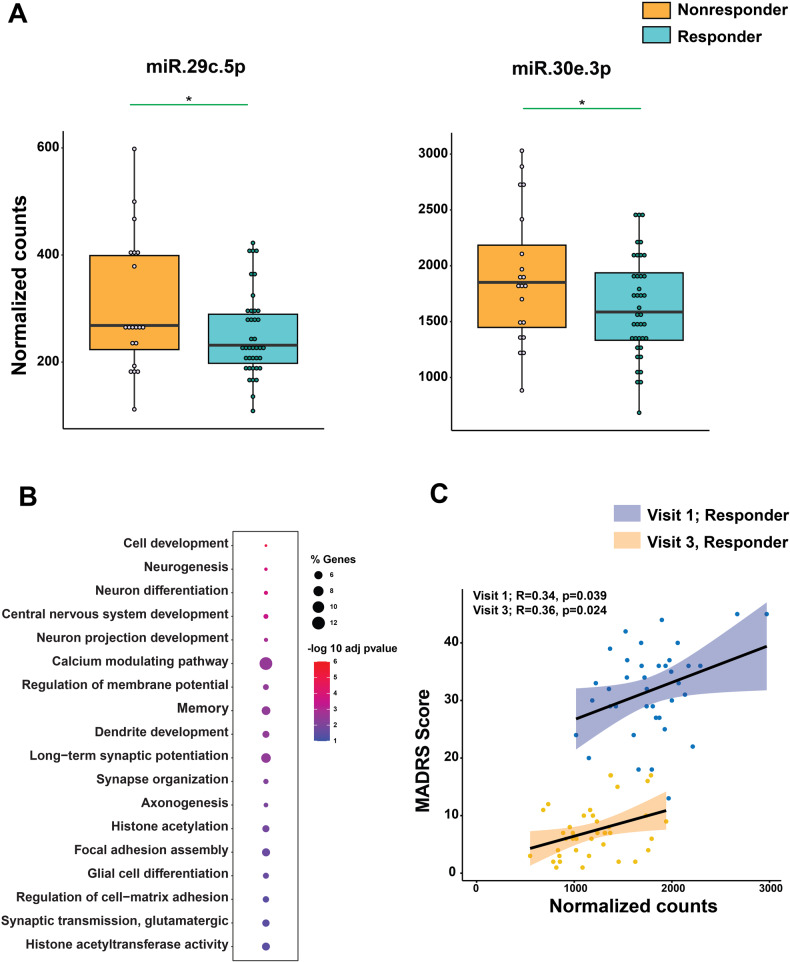
Peripheral blood miR change after completion of ECT treatment. This figure represents deregulated miRs in ECT-responder after completion of the course of ECT. **A** Significantly downregulated miRs in ECT responders. Y-axes represent normalized counts. Each dot in box plot represents normalized expression of miRs in each sample at visit 3. **B** Pathway analysis is showing deregulated biological processes (BP). Selected deregulated BP are enriched for neuron projection, neuron and glia development, cell adhesion, synapse and dendrite development and histone modifications. **C** Scatter plot of MADRS score and normalized counts at baseline (visit 1) and after completion of course of ECT. The correlation coefficient and its *p*-value were indicated by *r* and *p*, respectively. (*P < 0.05; **P < 0.01, ***P < 0.001; ****P < 0.0001). Two-tailed unpaired *t*-test was performed to calculate significance.

**Fig. 4:**
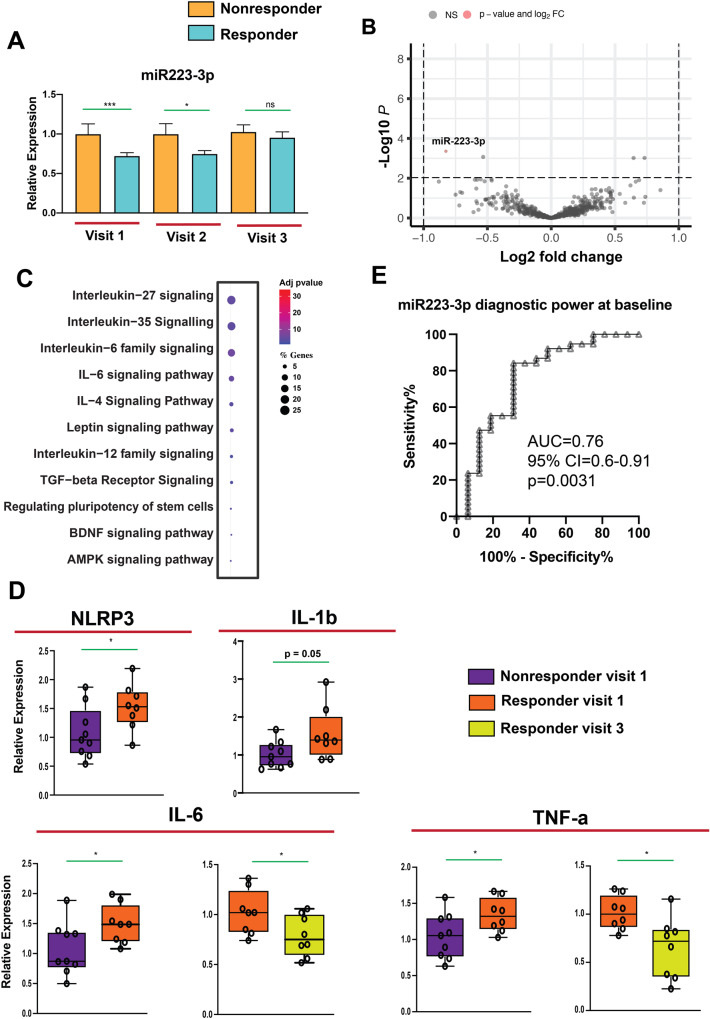
The immune system influence and contribute to the efficacy of ECT. **A** miR-223-3p expression in ECT-responder and non-responder subjects. Normalized counts of ECT- responders were compared with non-responders for each visit. Two-tailed unpaired t-test applied to evaluate the significance of expression difference between groups. The data are presented as the mean ± SEM in panel A. **B** The Volcano plot illustrates the relationship between the fold change and statistical significance at baseline for ECT responders and non-responders (visit 1). The negative log-fold change in the graph reflects miRs with downregulation, whereas the positive log-fold change represents miRs with up regulation. The only miR shown to be significantly dysregulated was miR-223- 3p (basemean ≥ 100, fold change ± 0.30, adjusted *p*-value < 0.1). **C** miRTarBase was used to detect validated targets of miR-223-3p. Pathways with adjusted *p*-value < 0.05 were selected for further analysis. miR-223-3p targeted genes are enriched for inflammation-related pathways in ECT responders. **D** The increase in NLRP3, IL-1b, IL-6 and TNF-α levels at baseline in responders compares to non-responders were observed. The baseline levels of IL-6 and TNF-α decrease towards the end of course of ECT among responders. (*P < 0.05 at the first and the last session; *t*-test unpaired; 2-tailed). The horizontal line in the box plot represents the median, the box spans 25 and 75% quantile, and the whiskers represent the smallest and largest values in the 1.5x interquartile range. **E** Receiver-operating characteristic (ROC) analysis of miR-223-3p in ECT-responder (*n* = 38) versus non-responder subjects (*n* = 16). Using z-score transformation of normalized counts, miR-223-3p had significant diagnostic power. (*P < 0.05; **P < 0.01, ***P < 0.001; ****P < 0.0001).

We compared the microRNAome of ECT responders and non-responders at visit 3 and found significant differential expression of miR-29c-5p and miR-30e-3p in responders (basemean ≥ 100, fold change ± 0.3; adjusted *p*-value < 0.05) (Fig. 3A; Supplementary fig. 4A, B). Both miRs decreased in responders after completing ECT treatment, despite not being differentially expressed at baseline (See Fig. 4 and supplementary table 8). These results suggest that ECT leads to a reduction of miR-29c- 5p and miR-30e-3p.

We analyzed the confirmed miR-29c-5p and miR-30e-3p targeted genes for GO biological processes associated with neuron differentiation, synapse organization, signal transduction, dendrite development, long-term potentiation, cell matrix adhesion, and histone acetylation (Fig. 3B). Furthermore, miR-29c-5p and miR-30e-3p were part of the MEyellow and MEred modules, respectively (Fig. 1C; Supplementary table 4). We performed a correlation analysis between miR-30e-3p and miR-29c-5p expression and MADRS score in ECT-responder subjects at baseline and after completion of ECT. Lower miR-30e-3p expression was positively correlated with symptom reduction and positive treatment response (baseline: *r* = 0.34, *p* = 0.039; After ECT: *r* = 0.34, *p* = 0.024) (Fig. 3C), but no significant correlation was found for miR-29c-5p. We also found significant overlap between miR-30e- 3p target genes and MDD associated genes (Supplementary fig. 4C). Our data suggest that ECT- induced changes in miR-30e-3p levels may affect biological processes related to synaptic plasticity and cell adhesion.

### miR-223-3p levels at baseline helps to predict therapeutic efficacy of ECT

We examined whether miR levels could predict ECT treatment response by analyzing differential expression between responders and non-responder at baseline. Only miR-223-3p showed significantly lower expression levels in ECT-responders compared to non-responders (basemean ≥ 100, fold change ± 0.3, adjusted *p*-value < 0.1; Fig. 4A, B, Supplementary table 8). This is in agreement with our finding that miR-223-3p is part of the red co-expression cluster (see Fig. 1C). We also analyzed changes in miR-223-3p expression during ECT treatment and found that the difference between responder and non-responder subjects decreased over time, with no significant difference at V3 (Fig. 4A; Supplementary table 9). Used target mining GO analysis we observed that miR-223-3p primarily regulates inflammation-related biological processes (Fig. 4C).

These findings support the hypothesis that altered levels of miR-223-3p reflect different immune function in ECT responders and non-responders [27]. Target mining revealed that miR-223-3p targets *interleukin 6* (*Il-6*)-6 and *NLR Family Pyrin Domain Containing 3* (*Nrlp3*), a key regulator of the inflammasome. Higher levels of IL-6, NLRP3, and TNF- α predicted a better response to ECT in patients with treatment-resistant depression [38–41]. Interestingly, baseline levels of *Il-6*, *Il-1b*, *Nrlp3*, and *Tnf- α* were higher in responder subjects (Fig. 4D). Since IL-6 is the known downstream target of the NLRP3 inflammasome, while TNF-α is an up-stream regulator, we compared *Il-6*and *Tnf-α* expression at baseline to post-treatment in responder subjects. Levels of *Il-6* and *Tnf-α* decreased from baseline to post-treatment in responder subjects (Fig. 4D; Supplementary table 10). These findings support the hypothesis that altered levels of miR-223-3p reflect different immune function in ECT responders and non-responders.

A ROC analysis showed that miR-223-3p has significant diagnostic power to distinguish ECT- responders from non-responders (Fig. 4E: AUC = 0.76, 95% CI = 0.6–0.91, *p* = 0.0031; Supplementary table 11). Additionally, a correlation analysis demonstrated a negative relationship between the difference in miR-223-3p expression and the percentage difference in MADRS score (*r* = −0.32, p = 0.04; Supplementary table 12), suggesting that low miR-223-3p expression at baseline correlates with high MADRS scores, while post-treatment increase in miR-223-3p expression is associated with reduced inflammatory response and depressive symptom improvement.

## Discussion

We examined small RNAome changes in whole blood associated with ECT responsiveness in patients with severe and treatment-resistant MDD. While several clinical factors such as higher age, psychotic symptoms, psychomotor disturbances, chronicity, or the number of previous pharmacological treatments have been linked to ECT response, the relationship between these factors and neurobiological changes is not yet clear [42–44].

We initially used a WGCNA approach to identify co-expressed miR modules associated with ECT response in our study. We found three modules that differed significantly between responders and non-responders, and their miR-target genes were enriched for various biological processes. Notably, we consistently observed several inflammation-related pathways in all three co-expression modules, as well as synaptic function-related pathways such as neuron-neuron synaptic transmission, dendrite development, and regulation of postsynaptic membrane potential. These findings are consistent with previous research on depression, which has shown that changes in inflammatory responses and synaptic function play a role in the pathogenesis and treatment of MDD [4, 11, 37, 45–49].

In addition to the WGCNA analysis, we found several miRs with altered expression levels between responders and non-responders following a single ECT or after completion of the therapeutic intervention. For instance, miR-30e-3p was significantly downregulated in ECT-responders following the last treatment and showed a positive correlation with MADRS scores when analyzed at baseline and after the last ECT. This is noteworthy since a polymorphism within the pre-miR-30e genes is associated with MDD risk [50]. In addition, miR-30e expression is increased in postmortem brain tissue from MDD patients that died of suicide and has been implicated with the regulation of the serotonergic system [51]. We also identified miR-223-3p as a potential predictor of ECT response at baseline, with lower expression levels in non-responders compared to responders and an AUC of 0.76. As ECT response is not guaranteed [52, 53], reliable predictors would be valuable for patient selection and treatment.

miR-223-3p was found to be differentially expressed in responders and non-responders, with its target genes linked to inflammation and interleukin-signaling pathways. This suggests that the patient’s inflammatory state may be responsible for the differential expression of miR-223-3p. Previous studies have linked miR-223-3p to the regulation of inflammatory cytokines and its increased expression was associated with reduced neuroinflammation and the regulation of inflammatory cytokines such as TNF-α, IL-1b, and IL-6 [54–58]. As well as an attenuation of depressive-like phenotypes in mice [59]. Furthermore, our analysis suggested that miR-223-3p may regulate NLRP3, a key component of the inflammasome that is activated via TNF-α signaling and controls IL-1b and IL-6 expression [60]. Inflammasome activation has been implicated in the pathogenesis of major depression [61].

In agreement with these data, ECT-responders have higher levels of *Nrlp3*, *Il-1b*, *Il-6*, and *Tnf-α* mRNA at baseline. This is consistent with other data showing higher proinflammatory cytokine levels in MDD patients prior to ECT [62, 63]. Moreover, decreased IL-6 and TNF-α levels are associated with hippocampal size and antidepressant efficacy [64, 65], highlighting the role of neuro-inflammation in depression and psychiatric disease [66, 67].

Interestingly, miR-223-3p levels were initially lower in responders, no difference was observed after ECT treatment, suggesting that ECT may affect miR-223-3p expression in patients with low levels. The results are consistent with other studies showing that effective treatment of depression leads to a normalization of cytokine levels [68–72]. Indeed, expression of IL-6 and TNF-α decreased in responders when comparing data from the first to the last ECT. However, further research is needed to fully understand the role of miR-223-3p and cytokines as biomarkers for ECT response and to determine if responders have a different neuroinflammatory state than non-responders.

Our study suggests that miR-223-3p could serve as a biomarker for ECT efficacy, but there are limitations to our work. Confounding factors such as body mass index tobacco and alcohol use, autoimmune disorders, and medication use can impact cytokine levels. However, concomitant medication was kept stable during ECT, making the included patients their own controls. It should be mentioned that another recent study analyzing miR expression in MDD patients undergoing ECT or Ketamine treatment found no difference of miR-223-3p levels between responders and non- responders at baseline, but employed a different experimental setting and a smaller cohort [24]. At present we cannot fully explain the discrepancy to our study and further research using larger sample sizes is needed to verify our results.

Both clinical features and ECT parameters may influence miR levels and ECT outcome. Besides a group difference regarding age (responders 58.79 ± 13.61 years, non-responders 50.3 ± 18,49 years; *p* = 0.042), we found a significant difference for stimulus dose (responders 94,56 ± 44.67 %, non- responders 61,91 ± 43,0 %; *p* = 0.007). However, age did not affect miR-223-3p levels and higher stimulus dose in responders can be explained by the increasing seizure threshold with higher age. Furthermore, the effectiveness of ECT does not so much depend on the electric current but rather on the induced seizure. Groups did not differ regarding EEG seizure duration (*p* = 0.725), and non- responders showed even better markers of seizure quality like PSI (*p* = 0.025) and ASEI (*p* = 0.03).

A key task remaining is to identify the source of dysregulated miRs in our data. To investigate this, cell- free smallRNA sequencing studies during ECT are essential. Unfortunately, we lacked the necessary plasma/serum samples. In this context it is important to note that brain-derived miRs have been shown to reach the circulation via extracellular vesicles [19, 73–75], suggesting that isolating microglia-derived extracellular vesicles from blood for analysis [21, 76, 77] could be an interesting future experiment.

This will also help to address the question if miRs such as miR-223-3p and the associated cytokines may serve as drug targets to treat MDD. For example, peripheral delivery of a TNF-α antagonist was shown to alleviate depression symptoms in patients with elevated inflammatory biomarkers [78, 79]. There is also emerging evidence for miRs as therapeutic targets in brain diseases and administration of miR mimics was shown to ameliorate depressive-like phenotypes in a rodent model for stress- induced depression [80].

In summary, we found differentially expressed miRs in MDD patients undergoing ECT, mainly linked to inflammatory processes. Our data also identify miR-223-3p as a potential regulator of inflammatory response which might prove helpful to distinguish responders from non-responder. These findings could help to develop paradigms for patient selection and improve ECT efficacy.

To conclude, ECT is a highly effective treatment for patients with pharmaco-resistant MDD which may exert its effects by affecting miR expression such as miR-223-3p leading to a reduction of inflammation. Still, further experiments are required to better comprehend the therapeutic processes of ECT and its effect on peripheral cytokines, particularly in individuals who do not respond to treatment.

### Supplementary information

Supplemental figures

Related Manuscript File

Related Manuscript File

Related Manuscript File

Related Manuscript File

Related Manuscript File

Related Manuscript File

Related Manuscript File

Related Manuscript File

Related Manuscript File

Related Manuscript File

Related Manuscript File

Related Manuscript File

## Data Availability

smallRNA sequencing data including metadata will be made available via the European Genome Phenome Archive or via the corresponding authors upon request.
