# Supplementary material for: Baseline levels of miR-223-3p correlate with the effectiveness of electroconvulsive therapy in patients with major depression
Source: Transl Psychiatry. 2023 Sep 13;13:294. doi: 10.1038/s41398-023-02582-4 (PMC10497550; doi:10.1038/s41398-023-02582-4)
Supplement: Supplementary file 1 — Supplemental figures [file 41398_2023_2582_MOESM1_ESM.pdf]

Supplementary Figure 1.

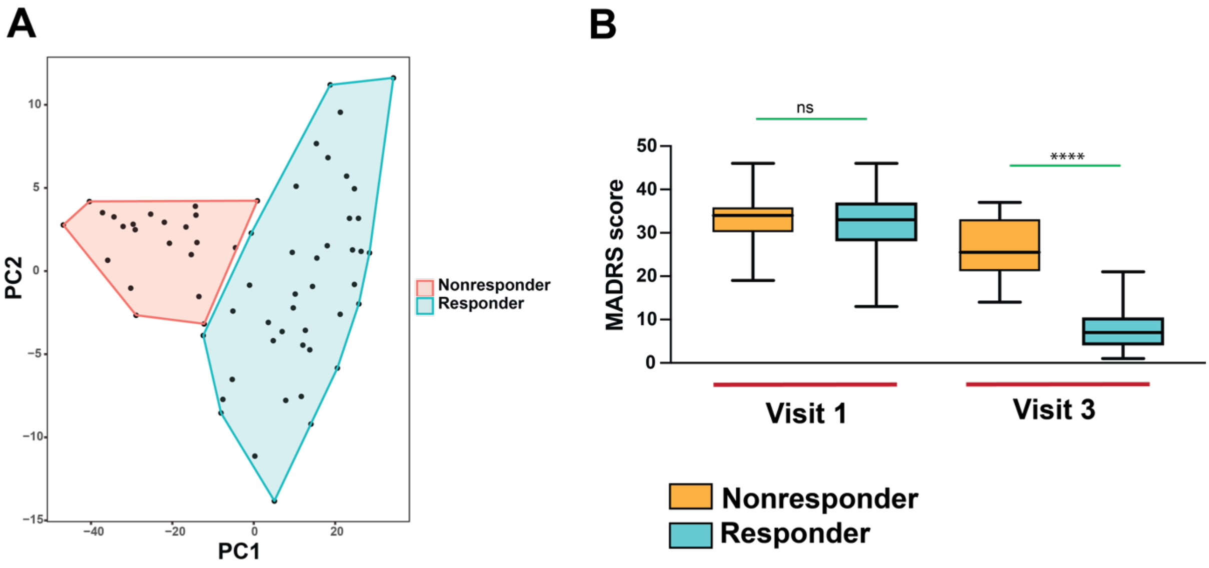

Supplementary figure 1: Characteristics of major depressive disorder (MDD) cohort

A) PCA plot depicting distribution of MADRS score difference before ECT and after the completion of course of ECT between responder and non-responder subjects. B) Depression rating score before and after ECT in ECT-responder and ECT-non responder group. Montgomery-Asberg Depression Rating Scale (MADRS) was used to evaluate the depression. Higher MADRS score indicates more severe depression. Linear regression model was applied to regress out the effect of age, gender if there is any. The horizontal line in the box plot represents the median, the box spans 25 and 75% quantile, and the whiskers represent the smallest and largest values in the 1.5x interquartile range. Mann-Whitney U tests were used to determine if MADRS scores differed significantly between ECT responders and non-responders. (*P < 0.05; **P < 0.01, ***P < 0.001; ****P < 0.0001).

Supplementary Figure 2.

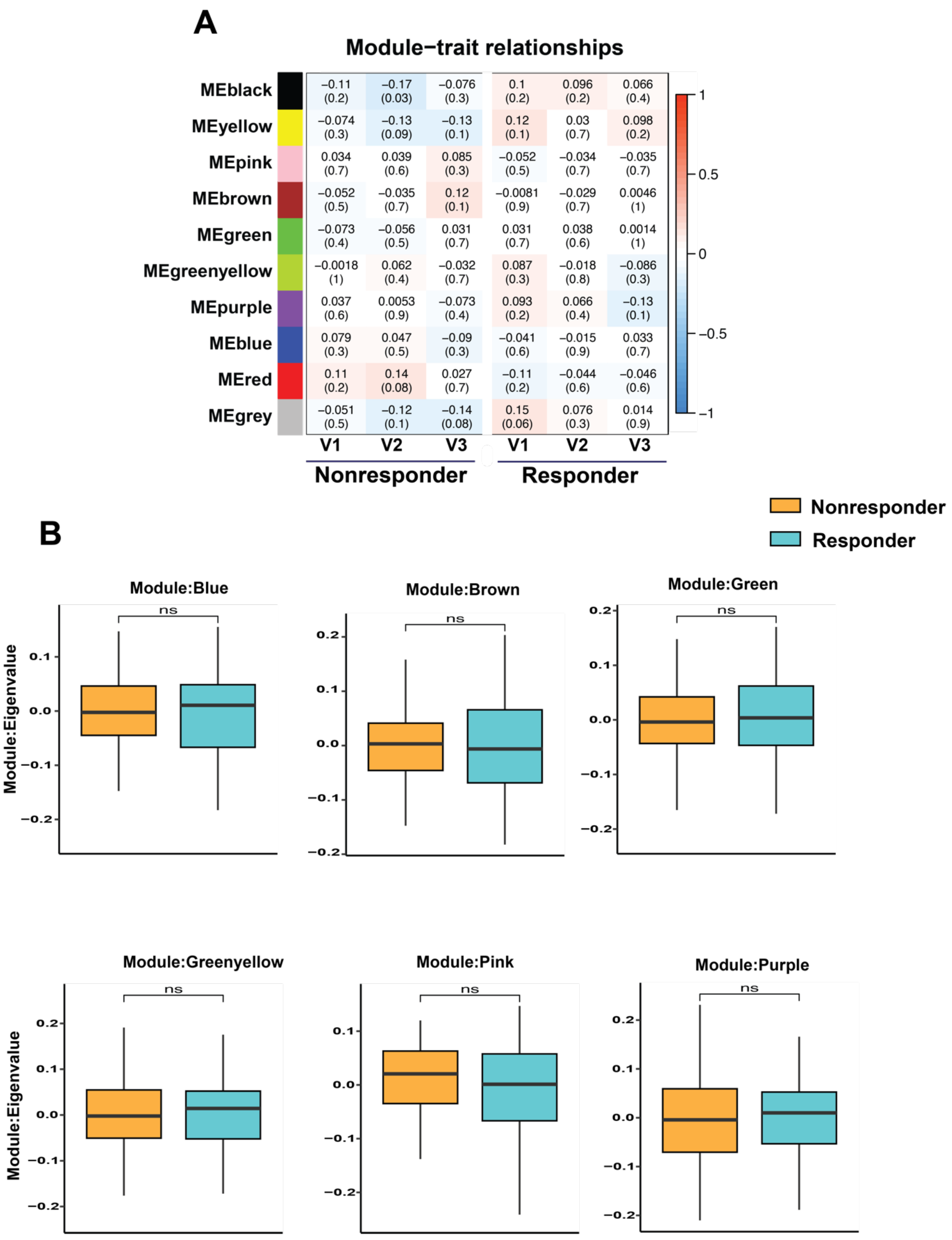

Supplementary figure 2: Co-expression analysis of microRNAome using WGCNA

A) Weighted co-expression analysis of microRNAome revealed co-expressed miRs in ECT-responder and non-responder group during the course of ECT (top: correlation coefficient; bottom: P-value). The intensity of the color indicates the extent of module correlation. Positive correlation is denoted by red, whereas negative correlation is denoted by blue. The correlation coefficient and P-value for each module are displayed. V1 represent visit 1 or baseline (before ECT started), V2 and V3 represent ECT treatment (total 60 subjects comprising 166 smallRNAseq dataset). B) Module eigengene values were plotted for remaining non-significant modules.

Supplementary Figure 3.

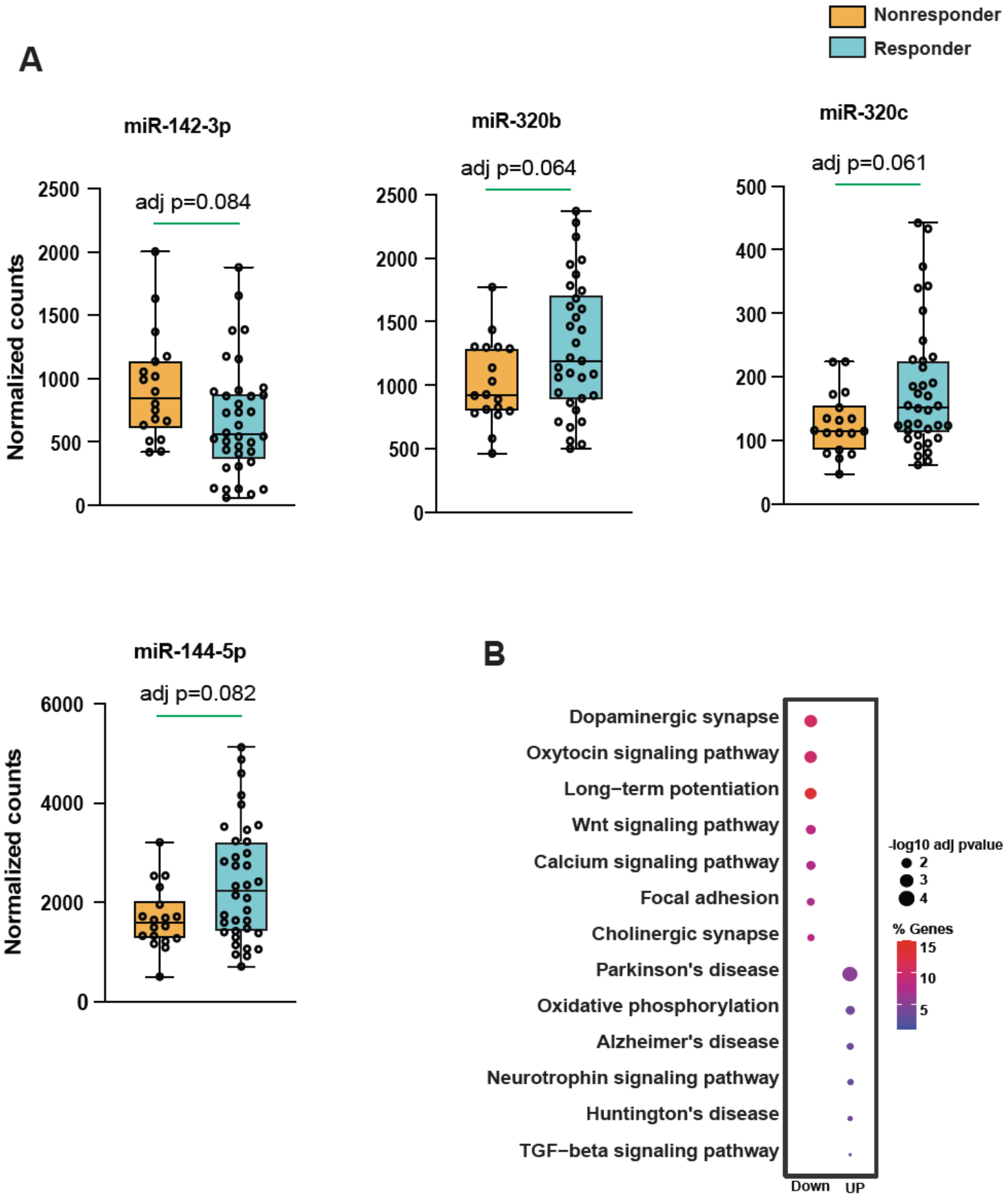

Supplementary figure 3: Effect of first treatment of ECT on microRNAome

This figure represents the analysis of deregulated microRNAs in ECT-responders after the first ECT treatment. A) Bar plots show the significantly deregulated microRNAs in ECT-responders. Y-axes represent normalized counts. Each dot in boxplot represents normalized expression of the corresponding microRNA in each sample. B) The dot plot shows the GO-term analysis (Biological processes) for target genes of the 3 up-regulated microRNAs (miR-320b, miR-320c and miR-144-5p;

up) and the target genes of the down-regulated miR-142-3p (down). (*$P < 0.05$; **$P < 0.01$, ***$P < 0.001$; ****$P < 0.0001$). Two-tailed unpaired t-test was performed to calculate significance

Supplementary Figure 4.

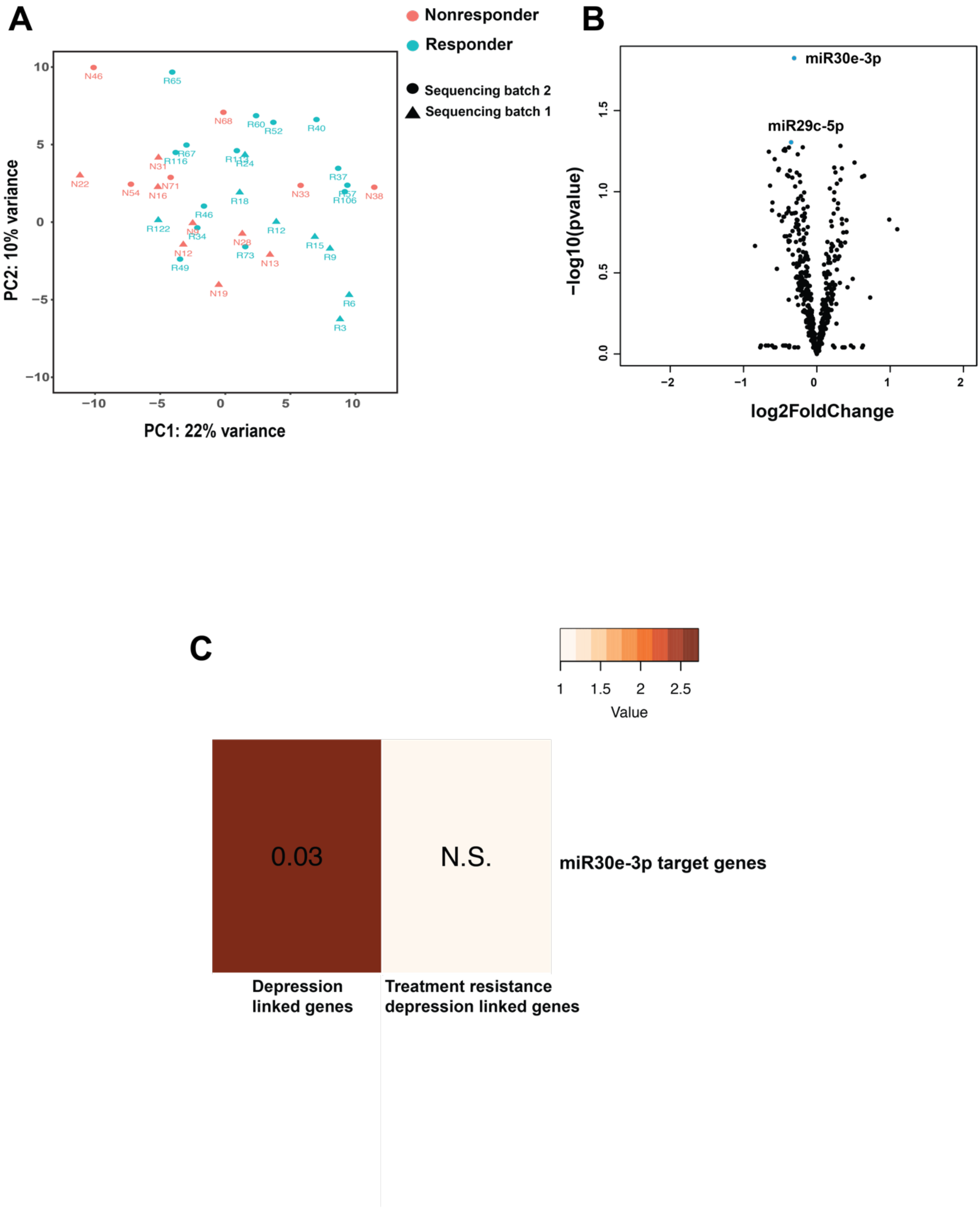

Supplementary figure 4: Deregulated miR expression after completion of ECT

A) PCA plot showing distribution of ECT-responder and non-responder after completion of course of ECT. B) Volcano plot shows the relationship between fold change and statistical significance. The blue points in the plot represent the significantly down regulated miRNAs with statistical significance. Significantly deregulated miRs are mentioned in the plot. C) Hypergeometric overlap between the

target genes of the miRs and genes associated with depression, as determined by human genome-wide association studies and transcriptional-based studies, is seen. Fisher's exact test was conducted, and the data are presented with a Benjamini Hochberg (BH) adjusted p value. Colour maps show the relevance and strength of the overlapping areas. We used the PsyGeNEt database to identify genes related with depression and TRD.
